# Editorial: Recent advances in oncolytic virus therapy for brain tumors

**DOI:** 10.3389/fcimb.2023.1271559

**Published:** 2023-08-17

**Authors:** Fatemeh Sana Askari, Alireza Mohebbi, Jianfang Ning, Kazuhiko Kurozumi, Hiroaki Wakimoto

**Affiliations:** ^1^ Department of Microbiology, School of Medicine, Golestan University of Medical Sciences, Gorgan, Iran; ^2^ Vista Aria Rena Gene Inc., Gorgan, Golestan, Iran; ^3^ Department of Virology, School of Medicine, Iran University of Medical Sciences, Tehran, Iran; ^4^ Department of Neurosurgery, University of Minnesota, Minneapolis, MN, United States; ^5^ Department of Neurosurgery, Hamamatsu Medical University, Shizuoka, Japan; ^6^ Department of Neurosurgery, Massachusetts General Hospital, Harvard Medical School, Boston, MA, United States

**Keywords:** oncolytic virus, glioblastoma, glioma, immunotherapy, tumor microenvironment

## Oncolytic viruses for the treatment of malignant gliomas

Malignant (or high-grade) glioma remains a significant medical problem due to devastating clinical outcomes and lack of effective therapeutic approaches. Glioblastoma (GBM) represents the most common and aggressive type of malignant gliomas, with median survival time 15-18 months despite the current standard of care consisting of surgery, radiation and alkylating chemotherapy. Owing to the capacity of cancer selective killing and induction of anti-tumor immunity, oncolytic viruses (OVs) have emerged as a strategy featuring unique mechanisms of action, offering the potential to address the unmet need in clinical neuro-oncology (Asija et al.). Experts in the field of OV participated in this Research Topic to highlight recent advances in preclinical and clinical development of OV therapies for GBM. A notable recent clinical advancement in the field is the conditional approval of the oncolytic herpes simplex virus (oHSV) Delytact (G47Δ) for the management of recurrent or progressive malignant glioma (Asija et al.; Kardani et al.). Currently, several OV platforms, both DNA and RNA viruses, are under active clinical investigation as monotherapies or combination therapies for GBM, with oHSV and oncolytic adenovirus being two of the most extensively studied virus types (Asija et al.; Liu et al.). Remarkably, GBM drives numerous mechanisms that contribute to tumor evasion of traditional treatments. Presence of GBM stem-like cells (GSCs) and suppressive immune cells in the tumor microenvironment are two key hallmarks of GBM that were the themes of Kardani et al. and Liu et al., respectively, in the context of OV therapy. Furthermore, Giehl et al. presented an original research work that elegantly demonstrated OV-induced priming of tumor specific effector T cells in a peritoneal cancer model, providing implications into glioma OV therapies.

### Targeting glioblastoma stem-like cells with oncolytic herpes simplex viruses

GSCs represent a distinct cell population within GBM that possess the abilities for self-renewal and differentiation into multiple cell types. These characteristics of GSCs contribute significantly to tumor progression, heterogeneity, resistance to therapy, and recurrence. Kardani et al. reviewed the potential of genetically engineered oHSVs for glioma treatment, with a specific focus on targeting GSCs. Generally, oHSVs exhibit desirable traits, including lytic nature, lack of integration into the host’s chromosomes, and susceptibility to antiviral drugs, making them an appealing candidate for targeting GSCs in the treatment of GBM. However, genetic modifications of oHSVs substantively impact their capacity to replicate in GSCs. Deletion of the neurovirulence gene γ34.5 found in clinical oHSVs renders oHSV highly attenuated in replication in GSCs. Delytact and rQNestin34.5v2 are designed to retain the capacity to eradicate GSCs without significantly increasing pathogenicity by immediate early expression of Us11 and re-insertion of γ34.5 under the control of GBM-selective nestin promoter, respectively (Kardani et al.; Liu et al.). The authors described various strategies to enhance the targeting and killing of GSCs using oHSVs, such as genetically modifying the viruses to express therapeutic transgenes (e.g., immunostimulatory cytokine IL-12), and combining oHSVs with other treatment modalities (e.g., DNA damage inducers, anti-angiogenic agents and immune modulatory agents). All of these are exciting approaches, but many are still at preclinical or early clinical developmental stage. The review underscores the need of continued research to improve oHSV efficacy and clinical outcomes, and highlighted current priorities that include developing potent but safe oHSVs, activating a potent anti-tumor immune response and identifying synergistic interactions with other agents.

### Overcoming the challenges of immunosuppressive cells in oncolytic virotherapy for glioma

The tumor microenvironment (TME) of GBM is highly immunosuppressive characterized by the abundance of immunosuppressive cells contributing to tumor progression and escape from therapies. Liu et al. focus on the challenges associated with immunosuppressive cells in OV therapy for GBM, as these cells in the immunosuppressive GBM microenvironment can play a major role in hampering the effectiveness of OVs. Immunosuppressive cells, such as tumor-associated microglia/macrophages (TAMs) and myeloid-derived suppressor cells (MDSCs), are present in this environment, diminishing anti-tumor T-cell mediated immune responses and restricting the therapeutic potential of OVs. Many OVs have been shown to be able to convert the phenotype of TAMs from T2 (suppressive) to T1 (inflammatory). Importantly, TAMs, the most abundant immune cells in the GBM TME, have the dual role in OV therapy; they mediate innate antiviral responses by secreting anti-viral cytokines such as TNFα and interferons and promote anti-tumor responses by inducing the recruitment of other effector immune cells and acting as antigen presenting cells. To address these challenges, several strategies have been explored, including combining OVs with immune checkpoint inhibitors and direct targeting of immunosuppressive cells, however, the timing of administration of TAM-depleting agents is likely critical for efficacy. The review emphasizes the importance of comprehending the intricate interactions between OVs and immunosuppressive cells, to fine-tune targeting of the immunosuppressive cells within the TME, to optimize therapeutic outcomes in glioma OV treatment.

### Boosting tumor-specific T cell responses with oncolytic viruses immunotherapy

Key feature of OV immunotherapy is elicitation of tumor specific T cell responses that contribute to overall efficacy. Giehl et al. utilized a vaccinia OV expressing the IL-15/IL-15Rα complex and demonstrated its potent induction of tumor-specific CD8+ T cells. Intraperitoneal delivery of the OV increased the levels of IL-15/IL-15Rα, cytotoxic CD8+ T cell responses, and the expression of cytotoxic proteins and Th1 cytokines within the TME. The treatment generated and enriched a tumor-specific CD8+ T cell population with cytotoxic and memory functions within the peritoneal cavity. These effector tumor-infiltrating immune cells were then harvested and used for adoptive cell therapy (ACT) in a murine model of peritoneal carcinomatosis, which resulted in increased animal survival and long-term responders. Thus, this work demonstrated the capacity of OVs expressing immunostimulatory molecules to prime and activate tumor-reactive T lymphocytes. Furthermore, the research outcome supports a potential of OV-stimulated autologous tumor-infiltrating lymphocytes (TILs) to be applied for effective ACT. Traditionally, ACT using TILs has not been effective in many solid cancers including GBM mostly due to the scarcity of autologous TILs with effector, killer functions. The OV-driven enhancement of producing tumor-specific effector T cells *in situ* might open a door to a neoadjuvant use of OV therapy for GBM followed by resection and TIL expansion for autologous ACT.

## Conclusion

**Figure 1:**
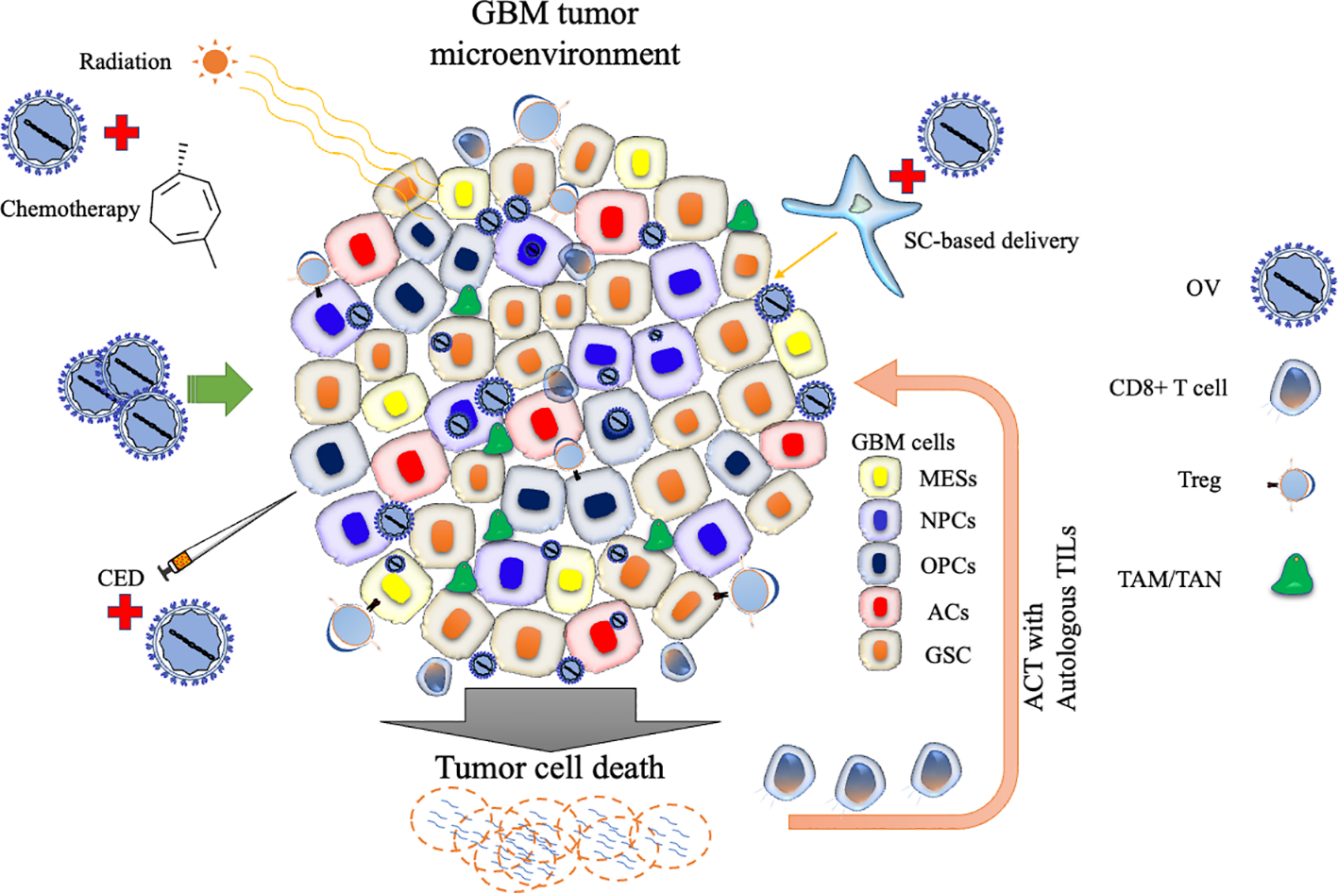
Schematic representation of oncolytic virus strategies targeting the GBM tumor microenvironment. The diagram highlights various cellular states and immunosuppressive cell types involved in glioblastoma (GBM) progression. Oncolytic virus (OV) therapy can be combined with conventional treatments such as radiation or chemotherapy to enhance efficacy. OV delivery can be improved by carrier cells or convention-enhanced delivery (CED). OV therapy can be used to generate cells used for adoptive cell therapy (ACT). Stem cell (SC); Tumor-associated macrophage (TAM); Tumor-associated neutrophil, TAN; Regulatory T cell (Treg); Tumor-infiltrating lymphocyte (TIL); Mesenchymal-like cell (MES); Neural progenitor-like cell (NPC); Oligodendrocyte progenitor-like cell (OPC); Astrocyte-like cell (AC); and glioblastoma stem-like cell (GSC).

The articles published in this Research Topic highlight the significant progress made in advancing OV immunotherapy for malignant glioma. The works overviewed several OV platforms that are currently actively investigated in clinical trials and demonstrated the potential of OVs to target GSCs, reprogram the suppressive TME, and enhance tumor-specific T cell responses to augment anti-tumor immunity and overall efficacy. Other critical areas for ongoing and future research include optimization of OV delivery and intratumoral spread and development of rational combination approaches (Asija et al.) (**Figure 1**). Rigorously designed clinical trials will tell us valuable findings as well as generate questions that may be brought back to the laboratories to provide answers. Such bedside to bench scientific crosstalk will advance OV therapy for malignant gliomas and ultimately help patients.

## Author contributions

FA: Writing – original draft, Writing – review & editing. AM: Writing – original draft, Writing – review & editing. JN: Writing – review & editing. KK: Writing – review & editing. HW: Writing – review & editing, Writing – original draft.

